# The Congress Programme—Important Correction

**Published:** 1893-05

**Authors:** 


### THE CONGRESS PROGRAMME—IMPORTANT CORRECTION.

THE editor of the *Dental Review* in the March number replies to a criticism of this journal, as follows:

"IMPORTANT CONGRESS NOTICE.

"The Editor of the INTERNATIONAL DENTAL JOURNAL is unfortunate in his interpretation of the printed order of business of the World's Columbian Dental Congress, recently sent out for publication. He objects to the daily 'Address before the whole Congress,' and says that 'The time given to addresses should be limited to the first day and the first session, and the other periods to scientific matters.'

"For fear others may fall into the same error of taking for granted that the daily addresses referred to are of a social character, we wish to make a plain statement to the effect that the matters treated on will be wholly of a scientific nature. The addresses will be from twenty to thirty minutes in length, and the subject matter will then be open for discussion before the whole Congress.

"The essential feature of these daily addresses consists in the requirement that they must be of general interest to the whole Congress, rather than of special interest to any one section.

"The Editor of the INTERNATIONAL DENTAL JOURNAL need not lament the lack of attention given to scientific work, for all the indications point to the fact that there will be more work of scientific value done in that one week of the Congress than ever was done before in an equal time in the history of the profession."

While it is a satisfaction to have this correction from the Secretary-General of the Congress, we cannot feel that the editorial in question was "unfortunate," but must regard it as having had a special value in settling the question of the addresses before the Congress. The unfortunate part was in the original wording of the programme, which should have been so explicit that no misconstruction could have been possible.

